# Comparison of Palliative Care Delivery in the Last Year of Life Between Adults With Terminal Noncancer Illness or Cancer

**DOI:** 10.1001/jamanetworkopen.2021.0677

**Published:** 2021-03-04

**Authors:** Kieran L. Quinn, Peter Wegier, Therese A. Stukel, Anjie Huang, Chaim M. Bell, Peter Tanuseputro

**Affiliations:** 1 Department of Medicine, University of Toronto, Toronto, Ontario, Canada; 2 ICES, Toronto and Ottawa, Ontario, Canada; 3 Institute of Health Policy, Management, and Evaluation, University of Toronto, Toronto, Ontario, Canada; 4 Department of Medicine, Sinai Health System, Toronto, Ontario, Canada; 5 Clinical Epidemiology Program, Ottawa Hospital Research Institute, Ottawa, Ontario, Canada; 6 School of Epidemiology, Public Health, and Preventive Medicine, University of Ottawa, Ottawa, Ontario, Canada; 7 Bruyère Research Institute, Ottawa, Ontario, Canada; 8 Department of Medicine, University of Ottawa, Ottawa, Ontario, Canada; 9 Interdepartmental Division of Palliative Care, Sinai Health System, Toronto, Ontario, Canada; 10 Temmy Latner Centre for Palliative Care, Toronto, Ontario, Canada

## Abstract

**Importance:**

Palliative care improves health outcomes, but studies of the differences in the delivery of palliative care to patients with different types of serious illness are lacking.

**Objective:**

To examine the delivery of palliative care among adults in their last year of life who died of terminal noncancer illness compared with those who died of cancer.

**Design, Setting, and Participants:**

This population-based cohort study used linked health administrative data of adults who received palliative care in their last year of life and died between January 1, 2010, and December 31, 2017, in Ontario, Canada.

**Exposures:**

Cause of death (chronic organ failure, dementia, or cancer).

**Main Outcomes and Measures:**

Components of palliative care delivery, including timing and location of initiation, model of care, physician mix, care settings, and location of death.

**Results:**

A total of 145 709 adults received palliative care (median age, 78 years; interquartile range, 67-86 years; 50.7% female); 21 054 died of chronic organ failure (4704 of heart failure, 5715 of chronic obstructive pulmonary disease, 3785 of end-stage kidney disease, 579 of cirrhosis, and 6271 of stroke), 14 033 died of dementia, and 110 622 died of cancer. Palliative care was initiated earlier (>90 days before death) in patients with cancer (32 010 [28.9%]) than in those with organ failure (3349 [15.9%]; absolute difference, 13.0%) or dementia (2148 [15.3%]; absolute difference, 13.6%). A lower proportion of patients with cancer had palliative care initiated in the home (16 088 [14.5%]) compared with patients with chronic organ failure (6904 [32.8%]; absolute difference, −18.3%) or dementia (3922 [27.9%]; absolute difference, −13.4%). Patients with cancer received palliative care across multiple care settings (92 107 [83.3%]) more often than patients with chronic organ failure (12 061 [57.3%]; absolute difference, 26.0%) or dementia (7553 [53.8%]; absolute difference, 29.5%). Palliative care was more often delivered to patients with cancer (80 615 [72.9%]) using a consultative or specialist instead of a generalist model compared with patients with chronic organ failure (9114 [43.3%]; absolute difference, 29.6%) or dementia (5634 [40.1%]; absolute difference, 32.8%). Patients with cancer (42 718 [38.6%]) received shared palliative care more often from general practitioners and physicians with subspecialty training, compared with patients with chronic organ failure (3599 [17.1%]; absolute difference, 21.5%) or dementia (1989 [14.2%]; absolute difference, 24.4%).

**Conclusions and Relevance:**

In this cohort study, there were substantial patient- and practitioner-level differences in the delivery of palliative care across distinct types of serious illness. These patient- and practitioner-level differences have important implications for the organization and scaled implementation of palliative care programs, including enhancement of practitioner education and training and improvements in equitable access to care across all settings.

## Key Points

**Question:**

How is palliative care being delivered to patients with noncancer illness (ie, chronic organ failure and dementia), and is it being delivered differently than for patients with cancer?

**Findings:**

In this cohort study, among 145 709 adults who died of cancer or terminal noncancer illness and received palliative care in their last year of life, palliative care was more likely to be initiated earlier, initiated in the hospital setting, and delivered across multiple care settings in patients with cancer than in those with organ failure or dementia. Patients with cancer were more likely to receive care using a consultative or specialist model instead of a generalist model and received palliative care more often from general practitioners and physicians with subspecialty training compared with patients with chronic organ failure or dementia.

**Meaning:**

Differences in the delivery of palliative care across distinct types of serious illness have important implications for the organization and scaled implementation of palliative care programs, including enhancement of practitioner education and training and improvements in equitable access to care across all settings.

## Teaser

This cohort study examines the delivery of palliative care among adults in their last year of life who died of terminal noncancer illness compared with those who died of cancer.

## Introduction

Palliative care improves quality of life, reduces symptom burden and unwanted health care use, and helps patients die in their preferred location.^1,2,3,4,5,6^ Palliative care is the active holistic care of people and their families experiencing suffering because of serious illness, especially near the end of life. It focuses on the prevention, early identification, assessment, and management of physical issues, including pain and other distressing symptoms, psychological distress, spiritual distress, and social needs.^7^ However, studies^1,4,8,9,10,11^ contrasting palliative care for patients with cancer and those with terminal noncancer illness report disparities in access and differences in the magnitude of its associated benefits.

A population-based study^9^ of 235 159 adults who died in Ontario, Canada, found that patients dying of cancer were more likely to receive palliative care than patients dying of noncancer illness, which included chronic organ failure and dementia. Relative to their death, patients with cancer were more likely to receive palliative care earlier than patients with noncancer illness. Prior population-based research^5,12^ demonstrated associated benefits of certain components of palliative care among 74 986 patients dying with heart failure (HF). Patients who received palliative care in the home from specialist palliative care practitioners had increased odds of a home death, a place where most patients prefer to die.

The focus of practitioners, decision makers, and health services researchers is shifting from an examination of the efficacy of palliative care across distinct types of serious illness to an examination of the successful design of palliative care programs to scale and implement them. Although prior research^13^ has evaluated differences in certain components of palliative care delivery for specific conditions (eg, HF), there is limited evidence investigating how the type of serious illness a patient has is associated with gaps in the delivery of multiple key components of palliative care. The current study is novel because it describes how multiple key elements of palliative care are being delivered across distinct types of serious illness at a population level with an aim to identify these gaps. The objective of this study was to describe the delivery of palliative care among adults in their last year of life who died of terminal noncancer illness (ie, chronic organ failure and dementia) compared with those who died of cancer.

## Methods

### Study Design, Setting, and Data Sources

We conducted a population-based cohort study in Ontario, Canada, using linked clinical and health administrative databases. Ontario is Canada’s most populous province, with more than 10 million adults. All residents of Ontario have access to hospital care and physicians’ services, and those 65 years or older are provided universal prescription drug insurance coverage. The administrative data sets used in this study were linked using encoded identifiers at the patient level at ICES (formerly the Institute of Clinical and Evaluative Sciences) (eAppendix 1 in the Supplement). ICES is an independent, nonprofit research institute whose legal status under Ontario’s health information privacy law allows it to collect and analyze health care and demographic data, without consent, for health system evaluation and improvement. These deidentified and encoded data sets are routinely used to conduct studies that involve palliative care.^4,5,9,14,15,16^ Ethics approval was obtained from Sinai Health System’s Research Ethics Board. This study followed the Reporting of Studies Conducted Using Observational Routinely-Collected Health Data (RECORD) reporting guidelines.^17^

### Study Cohort

Our cohort included all Ontario adults (≥18 years of age) who died of cancer or common terminal noncancer causes and received newly physician-delivered palliative care in their last year of life between January 1, 2010, and December 31, 2017. We identified the delivery of palliative care based on a set of unique physician claims fee codes (eAppendix 2 in the Supplement).^4,5,9,15,16,18,19^ These codes were created to specifically indicate the delivery of palliative care and are related to therapies not intended to be curative, such as symptom management or counseling. We excluded patients who received 2 or more palliative care claims in the year before their last year of life that indicated prior engagement with palliative care or received their first palliative care visit within 7 days of death because their practitioners would not likely have time to organize the necessary supports for a home death. We considered these people likely to be interested in engaging with palliative care in their approach to care. This new-user design is used in pharmacoepidemiologic studies and minimizes bias by restricting analysis to persons who are initiating treatment because these people are more likely to be similar at baseline when outcome risks are likely to vary over the time someone has been receiving treatment.^20^

### Types of Serious Illness

The study index date was 365 days before the patient’s death. The primary exposure was the patient’s type of serious illness, categorized according to the cause of death as cancer or noncancer illness. We defined terminal noncancer illness as HF, chronic obstructive pulmonary disease (COPD), end-stage kidney disease (ESKD), cirrhosis, stroke, and dementia because these diseases represent the most common terminal noncancer conditions.^1,4,5,6,16,21,22,23^ For the purposes of the analyses, we further divided types of serious illness into cancer, chronic organ failure (HF, COPD, ESKD, cirrhosis, or stroke), and dementia because these conditions are recognized as having unique trajectories of functional decline at the end of life that may influence a person’s palliative care needs.^9,21,22,23^ Cause of death was determined according to the *International Statisitcal Classification of Diseases and Related Health Problems—Tenth Revision, Canada (ICD-10-CA) *disease code on the patient’s death certificate.

After the identification of differences in the outcome of timing of palliative care initiation according to illness type, we conducted a prespecified, exploratory, hypothesis-generating analysis using the timing of palliative care relative to death as the exposure to measure the potential association of timing with location of death. The timing of palliative care was categorized according to commonly used periods in the published literature: 30 days or less, 31 to 90 days, and 91 days or more before death.^11,24,25,26,27,28,29,30,31^ Currently, there are no established standards on when to initiate palliative care.

### Patient Characteristics

We measured demographic and clinical variables, including age, sex, socioeconomic status, rural location of residence, comorbidities and chronic conditions,^32^ and hospital frailty risk score,^33^ using a 5-year look-back period. We also measured year of death, use of acute health care services in the 1 year before the study index date, and the timing of first palliative care consultation relative to death.

### Outcomes

The primary outcome was the timing of palliative care initiation, which we categorized according to commonly used time frames within the published literature (≤30 days, 31-90 days, and >90 days before death).^11,24,25,26,27,28,29,30,31^ The secondary outcomes were (1) location of initiation (clinic, home, hospital, subacute care, or case management), (2) model of care (generalist, consultive, or specialist palliative care [see below for description]), (3) physician mix (general practitioners and specialists), (4) care setting (outpatient, home based, inpatient, multiple locations, and case management), (5) total number of palliative care visits before death, and (6) location of death, which included home (including nursing homes and hospice), hospital (including intensive care unit), and other (eAppendix 3 in the Supplement). Deaths that occurred in 1 of Ontario’s estimated 4300 palliative care unit beds were categorized as other because they cannot be distinguished from other subacute care beds, such as those in a rehabilitation hospital, using administrative data.

Most palliative care is delivered by general practitioners in Ontario. We considered physicians to be palliative care specialists if their annual billing was comprised of more than 10% of palliative care fee codes, which is based on a previously validated method with a sensitivity of 76.0% and a specificity of 97.8%.^19^ Formal palliative care in Ontario is predominantly provided by physicians, nurse practitioners, registered nurses, social workers and personal support workers in hospitals, outpatient clinics, and at home. Patients generally require a referral from a physician to access specialized palliative care services but such services may be provided by nonpalliative care specialists without a referral.

We measured the delivery of 4 models of physician palliative care delivery.^5,34^ These models are derived using the proportion of palliative care fee codes claimed by physicians, which classifies them as a palliative care specialist or palliative care generalist as described above. The 4 models of palliative care were (1) no physician-based palliative care (0% of claims are palliative fee codes), (2) generalist palliative care (care is provided from a primary care physician or medical or surgical specialist, such as an oncologist or general surgeon, whose annual billing is composed of ≤10% of palliative care fee codes), (3) consultative palliative care (care provided by both palliative care specialists and generalists), and (4) specialist palliative care (a physician whose annual billing is composed of >10% of palliative care fee codes).

The setting of palliative care was determined using physician claims, which included the locations in which care was delivered. There were 5 possible settings where palliative services could be delivered: (1) clinic, (2) home, (3) inpatient setting, (4) multiple locations, and (5) third-party case management settings.

### Statistical Analysis

Descriptive statistics were used to measure patient characteristics, the delivery of palliative care, and locations of death. The association between type of serious illness (using cancer as the comparator) and the timing of palliative care relative to death (using ≤30 days as the reference) and the timing of palliative care (using ≤30 days as the comparator) and the location of death (using home as the reference) were estimated using a multinomial logistic generalized estimating equation approach. After the identification of differences in the outcome of timing of palliative care initiation according to illness type, we conducted a prespecified, exploratory, hypothesis-generating analysis using the timing of palliative care relative to death as the exposure to measure the potential association of the timing with the location of death. We stratified location of death outcomes by type of serious illness because we did not intend to directly compare the magnitude of the associations given that they are confounded by indication. Models were adjusted for age, sex, comorbidities, rurality, neighborhood income, hospital frailty risk score, and total number of hospitalizations in the 1 year before the study index date. We did not account for clustering by physician or facility because most people receive end-of-life care from many physicians in multiple care settings.

We used standardized differences to identify important differences for all secondary outcomes. Standardized differences (also known as the Cohen effect size index) of 0.2, 0.5, and 0.8 can be used to represent small, medium, and large effect sizes, respectively. An effect size index of 0.1 or less is considered to indicate good balance among variables.^35^ All analyses were performed using SAS statistical software, version 9.4 (SAS Institute Inc).

## Results

### Baseline Characteristics

**Figure 1.**
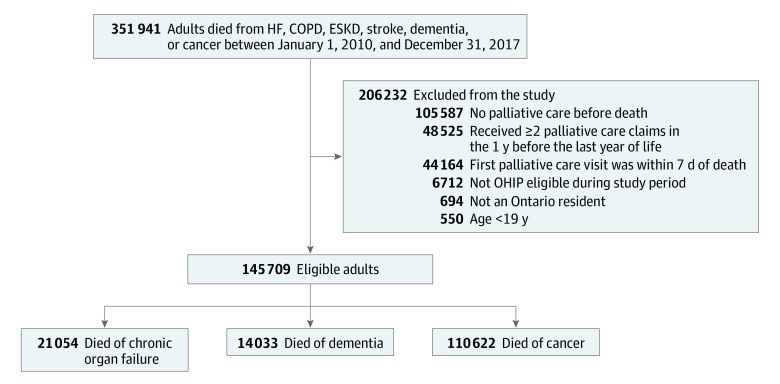
Flow Diagram for the Creation of the Study Sample All adults who died of heart failure (HF), chronic obstructive pulmonary disease (COPD), end-stage kidney disease (ESKD), stroke, dementia, or cancer were assessed for inclusion in the study. Patients who received their first consultation with palliative care within the last year of life at least 7 days before death were included. OHIP indicates Ontario Health Insurance Plan.

A total of 145 709 adults (median age, 78 years; interquartile range [IQR], 67-86 years; 50.7% female; 12.8% living in rural locations) received palliative care (Figure 1). The median hospital frailty risk score was 4 (IQR, 1-10) (Table 1). Among them, 21 054 died of chronic organ failure (4704 died of HF, 5715 of COPD, 3785 of ESKD, 579 of cirrhosis, and 6271 of stroke), 14 033 died of dementia, and 110 622 died of cancer.

**Table 1.** Baseline Characteristics of Patients Who Received Palliative Care in the Last Year of Life in Ontario Between 2010 and 2017 by Illness Type According to Their Cause of Death^a^

Characteristic	Cause of death			Standardized difference	
	Chronic organ failure^b^ (n = 21 054)	Dementia (n = 14 033)	Cancer (n = 110 622)	Chronic organ failure vs cancer	Dementia vs cancer
Age, median (IQR), y	85 (78-90)	88 (83-92)	74 (65-83)	0.89	1.47
Female sex	11 495 (54.6)	8993 (64.1)	53 341 (48.2)	0.13	0.32
Rural residence	2372 (11.3)	1048 (7.5)	15 176 (13.7)	0.07	0.20
Income quintile					
1	5173 (24.6)	3350 (23.9)	24 274 (21.9)	0.06	0.05
2	4554 (21.6)	3017 (21.5)	23 809 (21.5)	0	0
3	4061 (19.3)	2671 (19.0)	21 612 (19.5)	0.01	0.01
4	3635 (17.3)	2456 (17.5)	20 653 (18.7)	0.04	0.03
5	3550 (16.9)	2487 (17.7)	19 989 (18.1)	0.03	0.01
Missing	81 (0.4)	52 (0.4)	285 (0.3)	0.02	0.02
Hospital frailty risk score					
Mean (SD)	11.5 (8.9)	14.3 (9.8)	5.0 (6.0)	0.85	1.14
Median (IQR)	10 (5-17)	13 (7-20)	3 (0-7)	0.92	1.17
Chronic conditions					
Arrhythmia	7803 (37.1)	2782 (19.8)	15 107 (13.7)	0.56	0.17
Metastatic cancer	767 (3.6)	127 (0.9)	51 621 (46.7)	1.14	1.27
COPD	7122 (33.8)	1200 (8.6)	13 541 (12.2)	0.53	0.12
Congestive heart failure	8204 (39.0)	1884 (13.4)	8876 (8.0)	0.78	0.18
Coronary artery disease	5299 (25.2)	1717 (12.2)	12 974 (11.7)	0.35	0.02
Dementia	3786 (18.0)	8137 (58.0)	4834 (4.4)	0.44	1.42
Diabetes	6290 (29.9)	1922 (13.7)	24 560 (22.2)	0.26	0.13
Hypertension	18 434 (87.6)	11 593 (82.6)	75 771 (68.5)	0.47	0.33
Kidney disease	4545 (21.6)	1110 (7.9)	5967 (5.4)	0.49	0.10
Rheumatoid arthritis	812 (3.9)	403 (2.9)	2841 (2.6)	0.07	0.02
Stroke	5285 (25.1)	1514 (10.8)	6227 (5.6)	0.56	0.19
Prior health care use,^c^ median (IQR)					
No. of unique prescription medications	18 (11-25)	16 (10-22)	11 (0-19)	0.61	0.53
Emergency department visits^d^	2 (1-4)	1 (0-3)	2 (1-3)	0.09	0.38
Hospitalizations	1 (1-2)	1 (0-1)	1 (0-2)	0.21	0.34

Abbreviations: COPD, chronic obstructive pulmonary disease; IQR, interquartile range.

^a^ Data are presented as number (percentage) of patients unless otherwise indicated.

^b^ Chronic organ failure included heart failure, COPD, end-stage kidney disease, cirrhosis, and stroke.

^c^ Prior health care use in the 12 months before the last 6 months of life.

^d^ Emergency department visits not resulting in hospital admission.

### Delivery of Palliative Care

Palliative care was initiated earlier (>90 days before death) in patients with cancer (32 010 [28.9%]) than in those with organ failure (3349 [15.9%]; absolute difference, 13.0%; standardized difference, 0.3) or dementia (2148 [15.3%]; absolute difference, 13.6%; standardized difference, 0.3) (Table 2). After adjustment, patients dying of chronic organ failure had lower odds of palliative care initiation at 91 days or more (adjusted odds ratio [aOR], 0.48; 95% CI, 0.46-0.51) and between 31 and 90 days (aOR, 0.77; 95% CI, 0.75-0.80) relative to initiation at 30 days or less before death compared with patients with cancer. Patients dying of dementia had a lower odds of palliative care initiation at 91 days or more (aOR 0.42; 95% CI, 0.40-0.45) and between 31 and 90 days (aOR, 0.60; 95% CI, 0.57-0.62) relative to initiation at 30 days or less before death compared with patients with cancer (Table 3). Baseline characteristics of the cohort according to the timing of palliative care initiation are presented in eTable 1 in the Supplement. A lower proportion of patients with cancer had palliative care initiated in the home (16 088 [14.5%]) compared with patients with chronic organ failure (6904 [32.8%]; absolute difference, −18.3%; standardized difference, 0.4) or dementia (3922 [27.9%]; absolute difference, −13.4%; standardized difference, 0.3). Patients with cancer received palliative care across multiple care settings (92 107 [83.3%]) more often than patients with chronic organ failure (12 061 [57.3%]; absolute difference, 26.0%; standardized difference, 0.6) or dementia (7553 [53.8%]; absolute difference, 29.5%; standardized difference, 0.7). Overall, patients with cancer received more palliative care visits (median, 11 visits; IQR, 5-21 visits) than patients with chronic organ failure (median, 4 visits; IQR, 2-9 visits; standardized difference, 0.8) and dementia (median, 4 visits; IQR, 2-9 visits; standardized difference, 0.8) from initiation to death (Table 2).

**Table 2.** Delivery of Physician-Based Palliative Care and Location of Death for Patients Who Received Palliative Care in the Last Year of Life Who Died of Cancer and Noncancer Illness (Chronic Organ Failure or Dementia) in Ontario Between 2010 and 2017 by Cause of Death^a^

Variable	Cause of death			Standardized difference	
	Chronic organ failure^b^ (n = 21 054)	Dementia (n = 14 033)	Cancer (n = 110 622)	Chronic organ failure vs cancer	Dementia vs cancer
Timing of palliative care initiation relative to death, d					
>90	3349 (15.9)	2148 (15.3)	32 010 (28.9)	0.3	0.3
31-90	8543 (40.6)	5102 (36.4)	43 679 (39.5)	0.0	0.1
≤30	9162 (43.5)	6783 (48.3)	34 915 (31.6)	0.3	0.4
Location of first palliative care visit					
Clinic	7751 (36.8)	4313 (30.7)	44 756 (40.5)	0.1	0.2
Home	6904 (32.8)	3922 (27.9)	16 088 (14.5)	0.4	0.3
Hospital	2728 (13.0)	1348 (9.6)	16 942 (15.3)	0.1	0.2
Subacute care	204 (1.0)	198 (1.4)	736 (0.7)	0.0	0.1
Case management	3467 (16.5)	4252 (30.3)	32 087 (29.0)	0.3	0.0
Model of palliative care^c^					
Generalist	11 940 (56.7)	8399 (59.9)	30 007 (27.1)	0.6	0.7
Consultative	5185 (24.6)	3043 (21.7)	50 985 (46.1)	0.5	0.5
Specialist	3929 (18.7)	2591 (18.5)	29 630 (26.8)	0.2	0.2
Palliative care physician mix					
General practitioners only	15 751 (74.8)	11 256 (80.2)	59 704 (54.0)	0.5	0.6
General practitioner and subspecialists	3599 (17.1)	1989 (14.2)	42 718 (38.6)	0.5	0.6
Subspecialists only	1704 (8.1)	788 (5.6)	8200 (7.4)	0.0	0.1
Care setting					
Outpatient	3397 (16.1)	1800 (12.8)	7685 (6.9)	0.3	0.2
Home based	3643 (17.3)	1862 (13.3)	3747 (3.4)	0.5	0.4
Inpatient	538 (2.6)	339 (2.4)	1672 (1.5)	0.1	0.1
Multiple locations	12 061 (57.3)	7553 (53.8)	92 107 (83.3)	0.6	0.7
Case management^d^	1415 (6.7)	2479 (17.7)	5411 (4.9)	0.1	0.4
No. of palliative care visits, median (IQR)	4 (2-9)	4 (2-9)	11 (5-21)	0.8	0.8
Location of death^e^					
Home	11 232 (53.3)	10 520 (75.0)	69 208 (62.6)	0.2	0.3
Hospital	6438 (30.6)	1976 (14.1)	30 130 (27.2)	0.1	0.3
Other	3384 (16.1)	1537 (11.0)	11 284 (10.2)	0.2	0.0

Abbreviation: IQR, interquartile range.

^a^ Data are presented as number (percentage) of patients unless otherwise indicated.

^b^ Chronic organ failure included heart failure, chronic obstructive pulmonary disease, end-stage kidney disease, cirrhosis, and stroke.

^c^ The 3 models of palliative care were (1) generalist palliative care (eg, from a primary care physician or medical specialists, such as internists and oncologists), (2) consultation palliative care (ie, care from both palliative care specialists and generalists), and (3) specialist palliative care.^19^

^d^ Case management typically includes telephone support, weekly case management, and outpatient case conference.

^e^ Location of death included home (including nursing homes and hospice), hospital (including intensive care unit), and other (eAppendix 2 in the Supplement).

**Table 3.** Association of Illness Type and Timing of Palliative Care Initiation Among Patients in the Last Year of Life Who Died of Cancer and Terminal Noncancer Illness (Chronic Organ Failure or Dementia) in Ontario Between 2010 and 2017

Timing of palliative care initiation relative to death	Cause of death, OR (95% CI)	
	Chronic organ failure vs cancer	Dementia vs cancer
>90 d vs ≤30 d		
Unadjusted	0.40 (0.38-0.42)	0.35 (0.33-0.36)
Adjusted^a^	0.48 (0.46-0.51)	0.42 (0.40-0.45)
>90 d vs 31-90 d		
Unadjusted	0.75 (0.72-0.77)	0.60 (0.58-0.63)
Adjusted^a^	0.77 (0.75-0.80)	0.60 (0.57-0.62)

Abbreviation: OR, odds ratio.

^a^ Models were adjusted for age, sex, comorbidities, rurality, neighborhood income, hospital frailty risk score, and total number of hospitalizations in the 1 year before the study index date.

The models of generalist and specialist palliative care along with the physician mix also differed between patients with distinct types of serious illness and are presented in Table 2 along with standardized differences for each distinct model. Palliative care was more often delivered to patients with cancer using a consultative or specialist (80 615 [72.9%]) instead of a generalist model compared with patients with chronic organ failure (9114 [43.3%]; absolute difference, 29.6%) or dementia (5634 [40.1%]; absolute difference, 32.8%). Patients with cancer (42 718 [38.6%]) received palliative care more often from both general practitioners and physicians with subspecialty training compared with patients with chronic organ failure (3599 [17.1%]; absolute difference, 21.5%; standardized difference, 0.5) or dementia (1989 [14.2%]; absolute difference, 24.4%; standardized difference, 0.6) (Table 2). The delivery of palliative care across all patients in the cohort according to the timing of initiation is presented in eTable 2 in the Supplement.

### Location of Death

**Figure 2.**
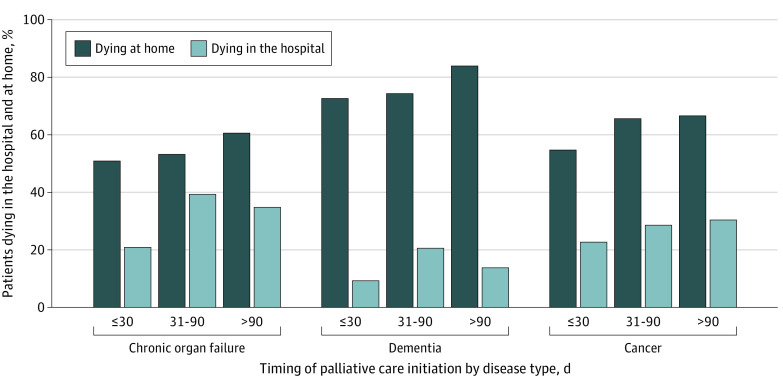
Location of Death Among Patients Receiving Palliative Care in the Last Year of Life in Ontario Between 2010 and 2017 According to Illness Type and Timing of Palliative Care Initiation

A higher proportion of patients with cancer (69 208 [62.6%]) died at home compared with patients with chronic organ failure (11 232 [53.3%]), but the proportion was lower than for patients with dementia (10 520 [75.0%]) (Figure 2). Differences were found in the magnitude of the association between timing of palliative care and location of death when comparing the different types of serious illness. After adjustment, no association was found with timing of first palliative care consultation (>90 days compared with ≤30 days before death) and the odds of dying in the hospital vs home (30.4% vs 22.7%; aOR, 0.98; 95% CI, 0.95-1.02) among patients with cancer. The timing of first palliative care consultation (>90 days compared with ≤30 days before death) was associated with higher odds of dying in the hospital for patients with chronic organ failure (34.8% vs 20.9%; aOR, 1.29; 95% CI, 1.18-1.42) and dementia (13.8% vs 9.3%; aOR, 1.28; 95% CI, 1.10-1.48) (Figure 2; eTable 3 in the Supplement).

## Discussion

This cohort study of 145 709 adults who died of cancer or noncancer illness and received palliative care in their last year of life found important differences in the delivery of palliative care across different types of serious illness for patients with cancer compared with those with chronic organ failure or dementia. In general, patients dying of cancer were younger, had a lower burden of medical comorbidity, and had lower prior health care use compared with patients dying of terminal noncancer illness. Palliative care was also initiated earlier and in the hospital or clinic setting for patients with cancer. For patients with cancer, palliative care was more often delivered by palliative care specialists across multiple locations. More patients with cancer also received palliative care from both general practitioners and physicians with subspecialty training compared with patients with chronic organ failure and dementia. These findings identify patient-level disparities in access to palliative care, which may be associated with the presence of established palliative care programs in cancer centers to provide multidimensional care for patients with cancer. These findings also suggest practitioner-level deficiencies in palliative care for patients with terminal noncancer illness that may be associated with differences in specialist self-rated knowledge of end-of-life care or recognition of their patient’s palliative needs and subsequent referral during their illness.^36,37,38,39,40,41,42^

The association between timing and location of death should be considered as exploratory, hypothesis-generating findings and interpreted with caution because of the risk of confounding by indication. A higher proportion of patients with chronic organ failure and dementia had palliative care initiated 30 days or less before death. This late initiation of palliative care in a population of patients with high medical complexity may limit sufficient time to build a trusting relationship with their palliative care practitioners and clarify their goals of care and preferences for location of death.^4,43^ Still, the fact that more patients with terminal noncancer illness died in the hospital despite having had palliative care initiated at home raises interesting questions that require further exploration because home palliative care is strongly associated with a home death.^5,15^ The fact that a much larger proportion of patients with cancer received palliative care in multiple settings (which includes home) may simply reflect differences in where patients are identified as having palliative care needs. Alternatively, this finding may be influenced by the availability and needs of family caregivers, especially for persons with dementia.

Our findings are supported by prior research^5,11^ demonstrating differences in the associated benefits of palliative care across types of serious illness. A population-based cohort study^11^ of 230 921 adults who died in Ontario found differences in the magnitude of association between the early initiation of palliative care (≥60 days before death) and health care use at the end of life across types of serious illness compared with late initiation (<60 days before death). In that study,^11^ early palliative care was associated with more than 2-fold higher odds of acute health care use at the end of life in patients dying of chronic organ failure and 1.5-fold odds in patients dying of dementia compared with acute health care use in patients dying of cancer. Prior research^5^ also examined differences in the associated benefits of different elements of palliative care delivery among 74 986 patients dying of HF. A subgroup analysis of the 35 292 patients who received palliative care found a strong association between palliative care initiated in the home, palliative care delivered across multiple settings, and models of care that involve specialist palliative care practitioners and a home death.^5^ The current study identified disparities among these components of delivery across different types of serious illness. Future work is required to measure the association of these differences with important outcomes and to identify differences in preferences for specific types of treatments between patients with different types of serious illness. Given that there are twice as many patients with noncancer illness and palliative care needs than there are with cancer, it is clear that there are not enough palliative care specialists to meet current or future demand.^44^ Health care systems should begin to focus on building capacity to integrate palliative care across all health care settings and levels (primary to tertiary care) throughout an illness, according to the patient’s needs. This work will require a commitment to delivering palliative care by all health care professionals with basic palliative care training, reserving specialist palliative care using multidisciplinary teams for referral of complex cases.^7^

### Limitations

This study has limitations. First, individual palliative care needs or specific palliative therapies were not measured among patients in the cohort. Patients with terminal noncancer illness frequently have a higher burden of palliative care needs associated with higher health care use, worse functional impairments, and higher levels of anxiety and depression compared with patients with cancer.^45,46,47^ In addition, patients with specific terminal noncancer illnesses, such as HF, ESKD, and cirrhosis, likely have differences in other care needs, such as ongoing dialysis, that occur during an exacerbation of their underlying disease. Second, this study described multiple differences in the components of palliative care as delivered by both generalist and specialist palliative care physicians. A stratified analysis of care delivery across illness trajectories comparing delivery by generalist and specialist palliative care practitioners would help identify further gaps in access to specialized palliative care and its associated benefits.^1,5,6,13,48^ However, a description of the real-world delivery of palliative care is important to identify gaps in care and likely strengthens the generalizability of the study to care provided in other similar health care systems. Third, the study did not measure delivery of palliative care by health care practitioners other than physicians, which may include nurse practitioners or social workers.^49,50^ Still, capturing the delivery of palliative care using physician fee codes in administrative data is a strength of the study given that care classification has been less successful in health systems without universal coverage.^51^ The prescription of medication (such as opioids, diuretics, and antipsychotics) may be an effective alternative or complementary approach to measuring the delivery of palliative care, including the timing of its initiation, beyond physician claims. Further work is required to address challenges associated with identifying the indication, dispensing, and continuous use of medications used with palliative intent. Fourth, the possibility that physicians were delivering palliative care and not using specific palliative care physician fee codes would serve to underestimate the magnitude of our findings. In Ontario, physicians are directed to use these codes for seriously ill patients who wish to forgo aggressive treatment of their underlying disease, with a focus on maintaining comfort. In practice, many physicians use the consensus definition from the International Association for Hospice and Palliative Care to determine if they are delivering palliative care.^7^ Fifth, the study intentionally used information on a patient’s death certificate to define the cohort to maximize specificity because of the concern that other approaches may introduce too much heterogeneity and other sources of bias.^4^

## Conclusions

There are substantial patient- and practitioner-level differences in the delivery of palliative care across distinct types of serious illness. These patient- and practitioner-level differences have important implications for the organization and scaled implementation of palliative care programs, including enhancement of practitioner education and training and improvements in equitable access to care across all settings.

## Notes

Supplement. **eAppendix 1.** Description of Datasets **eAppendix 2.** Physician Claims Fee Codes Used to Identify Delivery of Palliative Care **eAppendix 3.** Determining Location of Death **eTable 1.** Baseline Characteristics of Study Cohort by Cause of Death **eTable 2.** Delivery of Palliative Care to Patients in the Last Year of Life by Timing of Palliative Care Initiation **eTable 3.** Association of Timing of Palliative Care With Death in Hospital (Versus Home)
