# Error in Byline and Affilations

**DOI:** 10.1001/jamanetworkopen.2021.8238

**Published:** 2021-04-02

In the Original Investigation titled “Comparison of Palliative Care Delivery in the Last Year of Life Between Adults With Terminal Noncancer Illness or Cancer,”^1^ published March 4, 2021, Dr Wegier’s first name should have read Pete. His affiliations should have read as follows: Institute of Health Policy, Management, and Evaluation, University of Toronto, Toronto, Ontario, Canada; Humber River Hospital, Toronto, Ontario, Canada; and Department of Family and Community Medicine, University of Toronto, Toronto, Ontario, Canada.
