# Supplementary material for: Comparison of Palliative Care Delivery in the Last Year of Life Between Adults With Terminal Noncancer Illness or Cancer
Source: JAMA Netw Open. 2021 Mar 4;4(3):e210677. doi: 10.1001/jamanetworkopen.2021.0677 (PMC7933993; doi:10.1001/jamanetworkopen.2021.0677)
Supplement: Supplement. — eAppendix 1. Description of Datasets eAppendix 2. Physician Claims Fee Codes Used to Identify Delivery of Palliative Care eAppendix 3. Determining Location of Death eTable 1. Baseline Characteristics of Study Cohort by Cause of Death eTable 2. Delivery of Palliative Care to Patients in the Last Year of Life by Timing of Palliative Care Initiation eTable 3. Association of Timing of Palliative Care With Death in Hospital (Versus Home) [file jamanetwopen-e210677-s001.pdf]

Supplementary Online Content

Quinn KL, Wegier P, Stukel TA, Huang A, Bell CM, Tanuseputro P. Comparison of palliative care delivery in the last year of life between adults with terminal noncancer illness or cancer. *JAMA Netw Open*. 2021;4(3):e210677. doi:10.1001/jamanetworkopen.2021.0677

eAppendix 1. Description of Datasets
eAppendix 2. Physician Claims Fee Codes Used to Identify Delivery of Palliative Care
eAppendix 3. Determining Location of Death
eTable 1. Baseline Characteristics of Study Cohort by Cause of Death
eTable 2. Delivery of Palliative Care to Patients in the Last Year of Life by Timing of Palliative Care Initiation
eTable 3. Association of Timing of Palliative Care With Death in Hospital (Versus Home)

This supplementary material has been provided by the authors to give readers additional information about their work.

© 2021 Quinn KL et al. *JAMA Network Open.*

eText 1 - Description of datasets

All residents of Ontario have universal access to hospital care, physicians' services, and those aged ≥ 65 years of age are provided universal prescription drug insurance coverage without the requirement for co-payment. The administrative datasets used in this study were linked using encoded identifiers at the patient level and analyzed at ICES.

Database	Description
Canadian Institute for Health Information Discharge Abstract Database (CIHI-DAD)	Contains detailed diagnostic and procedural information for all hospital admissions in Canada.<br><br>DAD records have been demonstrated to have excellent agreement (over 99%) for demographic and administrative data. Regarding diagnoses, median agreement between original DAD records and re-abstracted records for the 50 most common most responsible diagnoses was noted to be 81% (Sensitivity 82%; Specificity 82%). The corresponding median agreement for the 50 most frequently performed surgical procedures was 92% (sensitivity 95%, positive predictive value 91%).[1]
Continuing Care Reporting System Long-Term Care (CCRS-LTC)	Contains demographic, administrative, clinical and resource utilization information on patients who receive continuing care services in hospitals or long-term care (LTC) homes in Canada. The long-term care dataset is generated from the Individual Assessment Instrument Minimum Data Set 2.0, a mandatory comprehensive, standardized and validated instrument for evaluating the needs, strengths, and preferences of elderly adults residing in nursing homes and receiving home care, contains detailed information on the functional status of these people.[2] Full assessments are completed on admission or referral, at quarterly intervals and following any significant health status change.
Home Care Database (HCD)	Contains patient-level data on government-funded home and community services.
National Ambulatory Care Reporting System (NACRS)	Reports demographic, administrative, clinical and service-specific data for Emergency Department visits.
National Rehabilitation Reporting System (NRS)	Contains patient data collected from participating adult inpatient rehabilitation facilities and programs across Canada
Ontario Congestive Heart Failure (CHF)	Contains all Ontario individuals with CHF identified since 1991.<br><br>A diagnosis of HF was identified by the presence of one hospital record or physician claim, followed by a second record from either source within 1 year. This

© 2021 Quinn KL et al. *JAMA Network Open.*

	method has been previously validated with a sensitivity of 84.8% and a specificity of 97.0%.[3]
Ontario Drug Benefit (ODB)	Provides individual prescription records including all prescriptions dispensed to Ontario residents aged 65 years and older. Each medication claim has an associated prescriber identifier which indicates the health practitioner who wrote the prescription.<br><br>An audit of 5,155 randomly selected prescriptions dispensed from 50 Ontario pharmacies determined that the ODB had an error rate of 0.7% and none of the pharmacy characteristics examined (locations, owner affiliation, productivity) were associated with coding errors.[4]
Ontario Health Insurance Plan (OHIP)	Identifies physician billing claims and specialty on all services provided by fee-for-service physicians in Ontario.
Ontario Mental Health Reporting System (OMHRS)	Documents data on patients in adult designated inpatient mental health beds. This includes beds in General, Provincial Psychiatric, and Specialty Psychiatric facilities.
Office of the Registrar General – Deaths (ORGD)<br><br><br><br><br>Registered Persons Database (RPDB)	An annual dataset containing information on all deaths registered in Ontario starting on January 1 1990 that includes the cause of death as indicated on their death certificate.<br><br>Registry of all Ontarians eligible to receive insured health services in the province and contains detailed demographic information as well as the Local Health Integration Networks (LHIN), which defines Ontario 14 regional areas within which people received most of their hospital care from local hospitals. The RPDB also provides information on the date and location of death for all individuals in Ontario.
Same Day Surgery (SDS)	Contains patient-level data for day surgery institutions in Ontario. Every record corresponds to one same-day surgery or procedure stay

eText 2 - Physician claims fee codes used to identify delivery of palliative care including location

Outpatient

- A945 (without and with B codes): Special palliative care consultation in clinic, office, home; minimum 50 min
- K015 (if no other feecode combination below was met): Counselling of relatives on behalf of catastrophically or terminally ill patient

© 2021 Quinn KL et al. *JAMA Network Open.*

- K023 (if no other feecode combination below was met): Palliative care support in half hour increments; may be used to add time for longer consultations following a code for A945, or for any PC support visit. Exclude if patient is in hospital, long-term care (LTC), complex continuing care (CCC), or rehabilitation

Home-based
- A900 with (B966, B998, B997): Complex house call assessment
- A901 with (B966, B998, B997): House call assessment
- A945 with any B code: Special palliative care consultation
- K023 with A900 A901 or any B code: Palliative care support
- K015 with A900 A901 or any B code: Counselling of relatives on behalf of catastrophically or terminally ill patient
- B966: Palliative care home visit; travel premium – weekdays daytime
- B998 : Palliative care home visit; special visit premium – weekdays daytime, first person seen
- B997: Palliative care home visit; special visit premium – nights, first person seen
- A900 A901 B960 B961 B962 B963 B964 B986 B987 B988 B990 B992 B993 B994 B996 within the last 3 months prior to death

Hospital inpatient
- C945: Special palliative care consultation
- C882: Palliative care; Non-emergency subsequent visits by the MRP following transfer from an Intensive Care Area
- C982: Palliative care; Emergency subsequent visits by the MRP following transfer from an Intensive Care Area
- K015 with (C945 C882 C982): Counselling of relatives on behalf of catastrophically or terminally ill patient
- K023 with (C945 C882 C982): Palliative care support in half hour increments; may be used to add time for longer consultations following a code for A945, or for any PC support visit.

Subacute care
- W882: Palliative care; Long-term care subsequent visit
- W982: Palliative care; Long-term care subsequent visit (for community medicine practitioners)
- K015 with (W882 W982): Counselling of relatives on behalf of catastrophically or terminally ill patient
- K023 with (W882 W982): Palliative care support in half hour increments; may be used to add time for longer consultations following a code for A945, or for any PC support visit.

Third-party encounters
- G511: Telephone services to patient receiving PC at home (max. 2/week)
- G512: Weekly care case management from palliative primary care management (Monday–Sunday)
- K700: Palliative care outpatient case conference

eText 3 - Determining location of death using RPDB

Hospital
- Hospital
- ICU

Home
- Community
- LTC

Other
- Subacute Care
- Unknown

© 2021 Quinn KL et al. *JAMA Network Open.*

eTable 1 – Baseline characteristics of patients receiving palliative care in the last year of life who died of cancer and terminal noncancer illness (chronic organ failure or dementia) in Ontario between 2010 and 2017 by timing of palliative care initiation.

	Timing of Palliative Care Initiation		
	>90 days prior to death (n=50,860)	>30 and ≤90 days prior to death (n=57,342)	≤30 days prior to death (n=37,507)
Age, years, median (IQR)	74 (64-83)	78 (68-86)	80 (70-87)
Female sex, n (%)	18,843 (50.2%)	29,389 (51.3%)	25,597 (50.3%)
Cause of Death			
Cancer	32,010 (85.3%)	43,697 (76.2%)	34,915 (68.6%)
Chronic Organ Failure	3,349 (8.9%)	8,543 (14.9%)	9,162 (18.0%)
Dementia	2,148 (5.7%)	5,102 (8.9%)	6,783 (13.3%)
Rural Residence, n (%)	5,038 (13.4%)	7,270 (12.7%)	6,288 (12.4%)
Income Quintile, n (%)			
1	8,079 (21.5%)	12,830 (22.4%)	11,888 (23.4%)
2	7,921 (21.1%)	12,453 (21.7%)	11,006 (21.6%)
3	7,373 (19.7%)	11,130 (19.4%)	9,841 (19.3%)
4	7,110 (19.0%)	10,574 (18.4%)	9,060 (17.8%)
5	6,933 (18.5%)	10,195 (17.8%)	8,898 (17.5%)
Missing	91 (0.2%)	160 (0.3%)	167 (0.3%)
Hospital Frailty Score, n (%)			
Mean (SD)	5.30 ± 6.89	6.50 ± 7.48	8.06 ± 8.12
Median (IQR)	3 (0-8)	4 (1-10)	6 (2-12)
0	9,686 (25.8%)	11,291 (19.7%)	6,907 (13.6%)
0.1-8.9	17,822 (47.5%)	27,361 (47.7%)	24,201 (47.6%)
9+	7,488 (20.0%)	14,504 (25.3%)	16,882 (33.2%)
Not hospitalized	2,511 (6.7%)	4,186 (7.3%)	2,870 (5.6%)
Chronic Conditions			
Arrhythmia	5,328 (14.2%)	10,020 (17.5%)	10,344 (20.3%)
Cancer			
Primary	11,842 (31.6%)	14,904 (26.0%)	11,799 (23.2%)
Metastatic	13,692 (36.5%)	19,970 (34.8%)	18,853 (37.1%)
COPD	4,919 (13.1%)	8,731 (15.2%)	8,213 (16.1%)
Congestive heart failure	3,680 (9.8%)	7,563 (13.2%)	7,721 (15.2%)
Coronary artery disease	4,612 (12.3%)	7,824 (13.6%)	7,554 (14.9%)
Dementia	2,581 (6.9%)	5,810 (10.1%)	8,366 (16.4%)
Diabetes	8,020 (21.4%)	13,204 (23.0%)	12,548 (24.7%)
Hypertension	25,466 (67.9%)	41,858 (73.0%)	38,474 (75.6%)
Renal disease	2,246 (6.0%)	4,426 (7.7%)	4,950 (9.7%)
Rheumatoid arthritis	1,000 (2.7%)	1,584 (2.8%)	1,472 (2.9%)

© 2021 Quinn KL et al. *JAMA Network Open.*

Stroke	2,382 (6.4%)	4,412 (7.7%)	6,232 (12.3%)
Prior healthcare use[a], median (IQR)			
No. unique prescription medications	12 (0-19)	13 (5-20)	13 (6-21)
Emergency department visits	2 (1-3)	2 (1-3)	2 (1-4)
Hospitalizations	1 (0-2)	1 (0-2)	1 (1-2)

[a]Prior healthcare use in the 12 months prior to the last 6 months of life
IQR – Interquartile range

© 2021 Quinn KL et al. *JAMA Network Open.*

eTable 2 – Delivery of palliative care to patients in the last year of life who died of cancer and terminal noncancer illness (chronic organ failure or dementia) in Ontario between 2010 and 2017 by timing of palliative care initiation.

	Timing of Palliative Care Initiation		
	**>90 days prior to death (n=37,507)**	**>30 and ≤90 days prior to death (n=57,342)**	**≤30 days prior to death (n=50,860)**
**Location of First Palliative Care Visit, n (%)**			
Clinic	16,780 (44.7%)	20,408 (35.6%)	19,633 (38.6%)
Home	3,247 (8.7%)	15,378 (26.8%)	8,292 (16.3%)
Subacute Care	145 (0.4%)	369 (0.6%)	633 (1.2%)
Hospital	3,285 (8.7%)	7,037 (12.3%)	10,696 (21.0%)
Case Management	14,050 (37.5%)	14,150 (24.7%)	11,606 (22.8%)
**Model of Palliative Care[a], n (%)**			
Generalist	9,644 (25.7%)	19,947 (34.8%)	20,755 (40.8%)
Consultative	20,023 (53.4%)	24,839 (43.3%)	14,351 (28.2%)
Specialist	7,840 (20.9%)	12,556 (21.9%)	15,754 (31.0%)
**Palliative Care Physician Mix, n (%)**			
General Practitioners Only	16,794 (44.8%)	34,293 (59.8%)	35,624 (70.0%)
General Practitioner and Subspecialists	18,028 (48.1%)	19,582 (34.1%)	10,696 (21.0%)
Subspecialists Only	2,685 (7.2%)	3,467 (6.0%)	4,540 (8.9%)
**Care Setting, n (%)**			
Outpatient	2,645 (7.1%)	3,864 (6.7%)	6,373 (12.5%)
Home-Based	486 (1.3%)	5,539 (9.7%)	3,227 (6.3%)
Inpatient	188 (0.5%)	559 (1.0%)	1,802 (3.5%)
Multiple Locations	31,799 (84.8%)	44,465 (77.5%)	35,457 (69.7%)
Case Management[b]	2,389 (6.4%)	2,915 (5.1%)	4,001 (7.9%)
**Number of Palliative Care Visits, median (IQR)**	20 (8-31)	10 (4-18)	5 (2-9)

[a]The three models of palliative care were: (1) generalist palliative care (e.g., from a primary care physician or medical specialists such as internists and oncologists), (2) consultative palliative care (i.e. care from both palliative care specialists and generalists), and (3) specialist palliative care.[5]

[b]Case management typically includes telephone support, weekly case-management and outpatient case-conference.

IQR – Interquartile range

© 2021 Quinn KL et al. *JAMA Network Open.*

eTable 3 – Association of timing of palliative care with death in hospital (versus home) among patients in the last year of life who died of cancer and terminal noncancer illness (chronic organ failure or dementia) in Ontario between 2010 and 2017. Results are presented as odds ratio (95% confidence interval).

	Timing of Palliative Care Initiation		
	**>90 versus 30-90**	**>90 versus ≤30**	**30-90 versus ≤30**
Death in hospital for patients with chronic organ failure (versus home[a])			
Unadjusted	0.77 (0.71-0.84)	1.39 (1.27-1.52)	1.80 (1.68-1.93)
Adjusted[b]	0.72 (0.66-0.78)	1.29 (1.18-1.42)	1.81 (1.68-1.94)
Death in hospital for patients with dementia (versus home[a])			
Unadjusted	0.60 (0.52-0.69)	1.29 (1.11-1.49)	2.17 (1.95-2.41)
Adjusted[b]	0.58 (0.50-0.67)	1.28 (1.10-1.48)	2.21 (1.98-2.46)
Death in hospital for patients with cancer (versus home[a])			
Unadjusted	1.05 (1.02-1.09)	1.10 (1.06-1.14)	1.05 (1.01-1.08)
Adjusted[b]	0.98 (0.95-1.01)	0.98 (0.95-1.02)	1.00 (0.97-1.04)

[a]Locations of death include home (including nursing home deaths), hospital (including ICU) and unknown.
[b]Models were adjusted for age, sex, comorbidities, rurality, neighborhood income, hospital frailty risk score, and total number of hospitalizations in the one year prior to the study index date.

© 2021 Quinn KL et al. *JAMA Network Open.*

eReferences

1. Juurlink DN, Preyra C, Croxford R, et al. *Canadian Institute for Health Information Discharge Abstract Database: A Validation Study*.; 2006.

2. Mor V. A comprehensive clinical assessment tool to inform policy and practice: applications of the minimum data set. *Medical care*. 2004;42(4 Suppl):III50-9.

3. Schultz SE, Rothwell DM, Chen Z, Tu K. Identifying cases of congestive heart failure from administrative data: a validation study using primary care patient records. *Chronic diseases and injuries in Canada*. 2013;33(3):160-166.

4. Levy AR, O'Brien BJ, Sellors C, Grootendorst P, Willison D. Coding accuracy of administrative drug claims in the Ontario Drug Benefit database. *The Canadian journal of clinical pharmacology = Journal canadien de pharmacologie clinique*. 2003;10(2):67-71.

5. Brown CR, Hsu AT, Kendall C, et al. How are physicians delivering palliative care? A population-based retrospective cohort study describing the mix of generalist and specialist palliative care models in the last year of life. *Palliative medicine*. 2018;32(8):1334-1343. doi:10.1177/0269216318780223

© 2021 Quinn KL et al. *JAMA Network Open.*
